# Supplementary material for: Motility, Adhesion and c-di-GMP Influence the Endophytic Colonization of Rice by Azoarcus sp. CIB
Source: Microorganisms. 2021 Mar 8;9(3):554. doi: 10.3390/microorganisms9030554 (PMC7998248; doi:10.3390/microorganisms9030554)
Supplement: Supplementary file 1 [file microorganisms-09-00554-s001.pdf]

Supplementary material

Motility, adhesion and c-di-GMP levels influence the endophytic colonization of rice by *Azoarcus* sp. CIB

Helga Fernández-Llamosas, Eduardo Díaz and Manuel Carmona *

Microbial and Plant Biotechnology Department. Centro de Investigaciones Biológicas Margarita Salas-CSIC. Ramiro de Maeztu 9, 28040 Madrid, Spain

* Correspondence: mcarmona@cib.csic.es

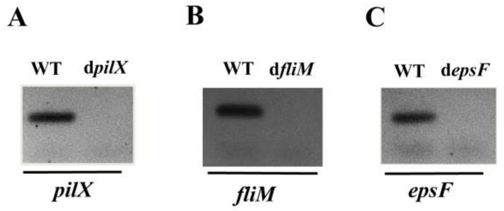

Figure S1. Expression of the *pilX*, *fliM* and *epsF* genes in the wild-type and mutant strains *Azoarcus* sp. CIB*dpilX*, *Azoarcus* sp. CIB*dfliM* and *Azoarcus* sp. CIBd*epsF*. (A) Electrophoresis in agarose gel of the RT-PCR product of the *pilX* gene obtained from RNA extracted from the *Azoarcus* sp. CIB (WT) and *Azoarcus* sp. CIBd*pilX* (dpilX) grown in VM medium to an A_{600} of 0.6. RNA extraction and RT-PCR technique was performed following the protocol detailed in Materials and Methods, using the 5'RTpilX/3'RTpilX oligonucleotide pair for amplification of the *pilX* gene fragment (Table S2). (B) Electrophoresis in agarose gel of the RT-PCR product of *fliM* gene obtained from total RNA isolated from strains of *Azoarcus* sp. CIB (WT) and *Azoarcus* sp. CIB d*fliM* grown in VM medium to an A_{600} of 0.6. RNA extraction and RT-PCR were performed following the protocol detailed in Materials and Methods, using the 5'RTfliM/3'RTfliM oligonucleotide pair for the amplification of *fliM* gene fragment (Table S2). (**C**) Gel electrophoresis of the RT-PCR product of the *epsF* gene obtained from total RNA extracted from the *Azoarcus* sp. CIB (WT) and *Azoarcus* sp. CIBd*epsF* (d*epsF*) grown in VM medium until an A_{600} of 0.6, using for amplification of *epsF* gene the oligonucleotides 5'RTepsF/3'RTepsF (Table S2).

Table S1. *Azoarcus* sp. CIB genes putatively involved in type IV *pili*, flagellum and exopolysaccharide production.

Function	Putative Genes Involved	Position in the Genome
Type IV *pili*	AzCIB_3113-3119 (*pil*)	3487 kb - 3494 kb
Type IV *pili*	AzCIB_3883-3887 (*pil*)	4334 kb – 4344 kb
Type IV *pili*	AzCIB_4164-4168 (*pil*)	4640 kb – 4645 kb
Flagellum	AzCIB_0942-0980 (*fli*/*flg*)	1060 kb – 1093 kb
Flagellum	AzCIB_3163-3166 (*flh*/*mot*)	3535 kb – 3538 kb
Flagellum	AzCIB_3565-3571 (*flh*/*mot*)	3984 kb – 3992 kb
Exopolysaccharide	AzCIB_0813-0833 (*eps*)	918 kb -942 kb
Exopolysaccharide	AzCIB_1833-1852 (*eps*)	2049 kb – 2071 kb
Exopolysaccharide	AzCIB_3666-3696 (*eps*)	4093 kb – 4134 kb

Adapted from [1]

Table S2. Oligonucleotides used in this study.

Primers	Sequence (5´to 3´)	Use
5′pilX	CGGGATCCCTGCTGGCCGTCTCCGCGATC (*Bam*HI)	451-bp *pilX* internal fragment cloned into double-digested pK18*mob* to generate pK18*mobpilX* used to construct CIBd*pilX* mutant
3′pilX	CCCAAGCTT-GTCATTTCCCACAGCGAAGTGG (*Hin*dIII)	451-bp *pilX* internal fragment cloned into double-digested pK18*mob* to generate pK18*mobpilX* used to construct CIBd*pilX* mutant
5′ext-pilX	AGGGTGCAGTCCTTTTCGT	Confirmation of the *Azoarcus* sp.CIBd*pilX* mutant
3′ext-pilX	CTCAGGTAGTCCAAGGCGTC	Confirmation of the *Azoarcus* sp. CIBd*pilX* mutant
5′fliM	CGGGATCCCCGACGAACCTGAAC-CTCATC (*Bam*HI)	508-bp *fliM* internal fragment cloned into double-digested pK18*mob* to generate pK18*mobfliM* used to construct CIBd*fliM* mutant
3′fliM	CCCAAGCTTAC-GTCGGCGCTGCCCAGATTG (*Hin*dIII)	508-bp *fliM* internal fragment cloned into double-digested pK18*mob* to generate pK18*mobfliM* used to construct CIBd*fliM* mutant
5′ext-fliM	CAACTACATGCACCGGAATG	Confirmation of the *Azoarcus* sp. CIBd*fliM* mutant
3′ext-fliM	ACCTTGATCGCGTAGTTCGT	Confirmation of the *Azoarcus* sp. CIBd*fliM* mutant
5′epsF	CGGGATCCGTCGTCGACGCGAAATCGG (*Bam*HI)	650-bp *epsF* internal fragment cloned into double-digested pK18*mob* to generate pK18*mobepsF* used to construct CIBd*epsF* mutant
3′epsF	CCCAAGCTTGAGATCGGCCTTCAGGTTCG (*Hin*dIII)	650-bp *epsF* internal fragment cloned into double-digested pK18*mob* to generate pK18*mobepsF* used to construct CIBd*epsF* mutant
5′ext-epsF	ATGAATCTGGGGCAATTCCTG	Confirmation of the *Azoarcus* sp. CIBd*epsF* mutant
3′ext-epsF	GTGCCTGGCTCTCCAGGCT	Confirmation of the *Azoarcus* sp. CIBd*epsF* mutant
F24	CGCCAGGGTTTTCCCAGTCACGAC	Check insertion mutant constructions in *Azoarcus* sp. CIB
R24	AGCGGATAACAATTTCACACAGGA	Check insertion mutant constructions in *Azoarcus* sp. CIB
5′RTpilX	CATACTGATCACGCTGCTGG	119-bp pilX fragment amplified in RT-PCR
3′RTpilX	GATCGAGGTGGTAATTCGCG	119-bp pilX fragment amplified in RT-PCR
5′RTpilY1	GGCGGTCATAGAAGAACTCG	80-bp *pilY1* fragment amplified in qRT-PCR
3′RTpilY1	TGCCCGAGGTATAAAGCATC	80-bp *pilY1* fragment amplified in qRT-PCR
5′RTfliC	GCGTGGATGCGTTTATTACC	97-bp fliC fragment amplified in qRT-PCR
3′RTfliC	TCGGTACCGTCAGCCTTG	97-bp fliC fragment amplified in qRT-PCR
5′RTfliM	TGTTCAACTACATGCACCGG	120-bp fliM fragment amplified in RT-PCR
3′RTfliM	GTTTGGCCAGGATGAGGTTC	120-bp fliM fragment amplified in RT-PCR

5’RTepsF	CCAGTATGCGCATTGGTTC	97-bp *epsF* fragment amplified in qRT-PCR and RT-PCR
3’RTepsF	GCCGTGCTTCTGCTGATAGT	97-bp *epsF* fragment amplified in qRT-PCR and RT-PCR
5’POLIIIHK	CGAAACGTCGGATGCACGC	166-bp *dnaE* fragment amplified in qRT-PCR
3’POLIIIHK	GCGCAGGCCTAGGAAGTCGAAC	166-bp *dnaE* fragment amplified in qRT-PCR

References:

1. Martín-Moldes Z, Zamarro MT, del Cerro C, Valencia A, Gómez MJ, Arcas A, *et al*. Whole-genome analysis of *Azoarcus* sp. strain CIB provides genetic insights to its different lifestyles and predicts novel metabolic features. Syst Appl Microbiol. 2015,38:462-71.
